# Cardiac responses to elevated seawater temperature in Atlantic salmon

**DOI:** 10.1186/1472-6793-14-2

**Published:** 2014-03-01

**Authors:** Sven Martin Jørgensen, Vicente Castro, Aleksei Krasnov, Jacob Torgersen, Gerrit Timmerhaus, Ernst Morten Hevrøy, Tom Johnny Hansen, Sissel Susort, Olav Breck, Harald Takle

**Affiliations:** 1 Nofima AS, P.O. Box 210, N-1431 Ås, Norway; 2 AVS Chile S.A., Casilla 300, Puerto Varas, Chile; 3 National Institute of Nutrition and Seafood Research (NIFES), P.O. Box 2029, Bergen, Nordnes, N-5817, Norway; 4 Institute of Marine Research, Matre Research Station, N-5984 Matredal, Norway; 5 Skretting Norway AS, P.O. Box 319, Sentrum N-4002 Stavanger, Norway; 6 Marine Harvest Norway AS, Sandviksbodene 78, N-5035 Bergen, Norway

**Keywords:** Temperature, Thermal acclimation, Cardiac tissue, Gene expression, Microarray, Immunofluorescence microscopy, iNOS, VEGF, Collagen I, Immune response

## Abstract

**Background:**

Atlantic salmon aquaculture operations in the Northern hemisphere experience large seasonal fluctuations in seawater temperature. With summer temperatures often peaking around 18-20°C there is growing concern about the effects on fish health and performance. Since the heart has a major role in the physiological plasticity and acclimation to different thermal conditions in fish, we wanted to investigate how three and eight weeks exposure of adult Atlantic salmon to 19°C, previously shown to significantly reduce growth performance, affected expression of relevant genes and proteins in cardiac tissues under experimental conditions.

**Results:**

Transcriptional responses in cardiac tissues after three and eight weeks exposure to 19°C (compared to thermal preference, 14°C) were analyzed with cDNA microarrays and validated by expression analysis of selected genes and proteins using real-time qPCR and immunofluorescence microscopy. Up-regulation of heat shock proteins and cell signaling genes may indicate involvement of the unfolded protein response in long-term acclimation to elevated temperature. Increased immunofluorescence staining of inducible nitric oxide synthase in spongy and compact myocardium as well as increased staining of vascular endothelial growth factor in epicardium could reflect induced vascularization and vasodilation, possibly related to increased oxygen demand. Increased staining of collagen I in the compact myocardium of 19°C fish may be indicative of a remodeling of connective tissue with long-term warm acclimation. Finally, higher abundance of transcripts for genes involved in innate cellular immunity and lower abundance of transcripts for humoral immune components implied altered immune competence in response to elevated temperature.

**Conclusions:**

Long-term exposure of Atlantic salmon to 19°C resulted in cardiac gene and protein expression changes indicating that the unfolded protein response, vascularization, remodeling of connective tissue and altered innate immune responses were part of the cardiac acclimation or response to elevated temperature.

## Background

Environmental temperature has been termed the master abiotic factor which controls and limits all biochemical, physiological and life history activities in teleost fishes [1]. The thermal optimum for different species have been extensively studied, representing the temperature where the difference between routine and maximum metabolic rates is greatest; i.e. the aerobic scope is at its maximum [2]. For Atlantic salmon (*Salmo salar* L.), optimum temperature for growth in sea has been found to occur at 13-15°C [3], with upper critical temperatures around 22°C [4]. In response to natural temperature fluctuations outside of the thermal tolerance window, fish respond by behavioral, biochemical and physiological modifications in order to maintain cellular homeostasis and physiological performance [5,6]. As the key organ supplying oxygen and fuels to the circulatory system for energy production, the heart has a major role in the physiological plasticity and acclimation to different thermal conditions in fish, showing alterations in cardiorespiratory performance, myocardial morphology and expression and phosphorylation of structural genes and proteins [7-10]. The occurrence of a thermal optimum (*T*_opt_) for cardiovascular function is reflected by different salmonid species having different *T*_opt_ for maximum oxygen uptake, aerobic scope and critical swimming speed [11,12]. At temperatures below and above *T*_opt_ the scope for aerobic metabolism will decline until a critical temperature (*T*_crit_) is reached, where no aerobic activity can be performed besides routine metabolism [1]. In salmonids, the decreased aerobic scope observed with increasing temperatures above *T*_opt_ is associated with a limited oxygen supply suggested to be caused by a failure in maximum cardiac output to increase above *T*_opt_[13]. Acclimation to high temperatures has been associated with cardiac remodeling of tissue composition and morphology [10], which is assumed to compensate for the decreased power-generating ability [14]. The nitric oxide synthase (NOS) system is another important inter- and intracellular regulator of cardiac function and oxygen supply in fish [15], and in long-term warm acclimated eel (*Anguilla anguilla*) inhibition of NO production significantly reduced the Frank-Starling response [16]. Another interesting yet poorly understood aspect of cardiac responses to temperature increase in fish is the effects on hematological and immunological responses, which may have a significant impact on the health and disease performance of Atlantic salmon in aquaculture, since heart is a target organ for several harmful viral pathogens [17,18].

In Atlantic salmon aquaculture in the Northern hemisphere, fish are exposed to large seasonal fluctuations in seawater temperature. Peak summer temperatures around 18-20°C are regularly experienced at production sites in the western and southern regions of Norway, causing concerns regarding the possible negative impact on productivity, fish performance and welfare. We recently reported that long-term exposure (56 days, simulating a warm water period in aquaculture) of adult (~2 kg) Atlantic salmon to 19°C under controlled conditions significantly reduced growth performance when compared to fish reared at 14°C, a difference driven by a 50% reduction in feed intake [19]. The objective of the present study was to investigate effects of such temperature increase on molecular responses in cardiac tissues from the same experimental fish. To achieve this, cDNA microarray screening and single gene expression validation with real-time qPCR were employed to evaluate transcriptional changes after 21 days (simulating a short warm water period) and 56 days (simulating a long warm water period) thermal acclimation. In addition, expression of selected proteins of interest were analysed with immunofluorescence microscopy in cardiac tissues after long-term thermal acclimation.

## Methods

### Temperature challenge trial

The experimental design is described in detail elsewhere [19]. This study used half of the groups; those fed the standard diet (L34). In brief, 170 adult (~1.6 kg) immature Atlantic salmon of the Norwegian salmon procreation strain (NLA) were randomly selected from sea cages and distributed into six 5.3 m^3^ light-gray round tanks (3 m diameter × 0.75 m water depth, temperature 14°C) at Matre Research Station, Matre (61°N), Norway. After 50 days acclimation period all fish were weighed (average body weight 2.0 ± 0.4 kg), and the temperature in three of the tanks was increased to 19°C at a rate of 1°C per day, while the three remaining tanks were kept at 14°C. Fish were reared under simulated natural photoperiod in 35 g L^-1^ seawater and oxygen level was kept constant on 90% saturation (measured continuously in the water outlet) by adding oxygen-supersaturated seawater (350% saturation). Fish were fed by automatic feeders that were adjusted daily to maintain 10% in excess. Feed which were not eaten were collected in an outlet trap. Feed was offered between 8 and 9 am and between 1 and 2 pm. To standardize sampling, all fish were fed *ad libitum* exactly four hours before sampling. Individually sampled fish (3 per tank, N = 9) were killed by a blow to the head and weights and fork lengths were measured to the nearest g and nearest 0.5 cm at the start, 21 days, and 56 days after commencement of the temperature increase. On days 0, 21 and 56, heart samples were collected from all sampled individuals under sterile conditions and divided in two; one half was flash-frozen in liquid nitrogen and stored at -80°C for gene expression analyses while the other half was fixed in 4% paraformaldehyde for immunofluorescence microscopy. The trial was approved by The National Animal Research Authority according to the ‘European Convention for the Protection of Vertebrate Animals used for Experimental and other Scientific Purposes’ (EST 123).

### RNA extraction

Sampled hearts for gene expression analyses were stored at -80°C prior to RNA extraction. Standardized tissue sections of 10 mg (equal mix of ventricle and atrium) were prepared under sterile/RNase-free conditions and transferred directly to 1 ml chilled TRIzol (Invitrogen, Carlsbad, CA, USA) in 2 ml tubes with screw caps (Precellys®24, Bertin Technologies, Orléans, France). Two steel beads (2 mm diameter) were added to each tube and the tissue was homogenized in a Precellys®24 homogenizer for two times 25 sec at 5000 rounds per minute with a break of 5 sec between rounds. RNA was extracted from the homogenized tissues using PureLink RNA Mini kits according to the protocol for TRIzol-homogenized samples (Invitrogen). The concentration of extracted total RNA was measured using NanoDrop 1000 Spectrometer (Thermo Scientific, Waltham, MA, USA), while RNA integrity was determined using Agilent 2100 Bioanalyzer with RNA Nano kits (Agilent Technologies, Santa Clara, CA, USA). Only samples with a RNA integrity number (RIN) of 8 or higher were accepted.

### Microarray analysis

Two microarrays were used for screening of transcriptional responses to high temperature (19°C) at both 21 and 56 days after temperature was raised from control (14°C). Each control and high temperature group consisted of a pool of 9 fish randomly selected from triplicate tanks per each time point. The salmonid fish cDNA microarray SFA2.0 (GEO Omnibus GPL6154) includes 1,800 genes, each printed in six spot replicates. Synthesis of cDNA and hybridizations were carried out as previously described [20]. In brief, samples with 10 μg RNA in each were labeled with Cy3-dUTP (reference control, 14°C groups) and Cy5-dUTP (test, 19°C groups) (Amersham Biosciences, UK) during cDNA synthesis using the SuperScript III reverse transcriptase kit (Invitrogen). After hybridization, slides were washed in 0.5 × SSC/0.1% SDS (15 min), 0.5 × SSC/0.01% SDS (15 min), and twice in 0.06 × SSC (3 min each) at room temperature in dim lighting with gentle agitation. Slides were dried using ArrayIt® Microarray High-Speed Centrifuge. Scanning was performed with GenePix 4100A microarray scanner (Molecular Devices, CA, USA) at 5 μm resolution and with manually adjusted laser power to ensure an overall intensity ratio close to unity between Cy3 and Cy5 channels, and with minimal saturation of features. Images were processed with GenePix Pro 6.0 software. Spots were filtered by criterion (I - B)/(SI + SB) ≥ 0.6, where I and B are mean signal and background intensities and SI and SB are standard deviations, respectively. Low-quality spots were excluded from analyses and genes with less than three high-quality spots on a slide were discarded. After subtraction of median background from median signal intensities and Lowess normalization, differential expression was assessed by difference of the mean log_2_-ER (expression ratios, high versus control temperature groups) from zero (six spot replicates per each gene; Student’s *t*-test, p < 0.01). Complete data are provided in the GEO Omnibus (accession number GSE53908). Genes with log_2_-ER > 0.4 in at least one time point and common functional annotation according to the STARS program [21] were considered for interpretation in the Results section.

### Quantitative real-time RT-PCR (qPCR)

Experiments were conducted according to the MIQE guidelines [22]. Synthesis of cDNA was performed on 0.2 μg DNAse-treated total RNA (Turbo DNA-free™, Ambion, Austin, TX, USA) using the TaqMan® Gold Reverse Transcription kit (Applied Biosystems, Foster City, CA, USA) in 25 μl reactions with random hexamer priming according to manufacturer’s protocol. Complementary DNA was stored undiluted at -80°C in aliquots to avoid repeated freeze-thawing. To avoid risk for presence of residual DNA contamination, control reactions without RT were tested and qPCR primers were designed to span introns when possible. Oligonucleotide primers for genes of Atlantic salmon were designed with the program eprimer3 from the EMBOSS program package (version 5.0.0, http://emboss.sourceforge.net/). Amplicon size was set to 80-200 and melting temperature to 59-61°C. Primers were purchased from Invitrogen (Table 1). *In silico* analysis of gene targets was performed using the STARS program for BLAST and sequence alignments. PCR amplicon size and specificity were confirmed by gel electrophoresis and melting curve analysis (Tm calling; LightCycler®480, Roche Diagnostics, Mannheim, Germany). QPCR was conducted in duplicate reactions as previously described [23]. Cycle threshold (C_T_) values were calculated using the fit point method. Duplicate measurements that differed more than 0.5 C_T_ values were removed and reanalyzed. For relative quantification, the mean of duplicates was used. Relative gene expression ratios of test samples versus the average of the normalized controls (14°C) were calculated according to the Pfaffl method [24] with normalization using the following reference genes: *NADH dehydrogenase (ubiquinone) 1 beta subcomplex 19 kDa* and *SEC13-like protein* (used for all genes except HSP70), or *elongation factor 1 alpha* (used for HSP70 only). All reference genes were validated using the BestKeeper software [25]. The efficiency of the PCR reactions was estimated for all primer pairs by six times 1:5 dilution series of a cDNA mix of all used samples. Efficiency values were estimated by using the LightCycler® 480 Software (version 1.5.0.39). Differences in gene expression ratios (log_2_-transformed values) between groups were assessed by two-sided pairwise t-tests with pooled standard deviation and p-value adjustment according to Holm (stats-package in R version 3.0.2 (http://cran.r-project.org).

**Table 1:** Genes and primer sequences used for qPCR analyses

**Gene**	**Dir.**	**Primer sequence (5′-3′)**	**GenBank acc. no.**
HSP70^1^	F	TGACGTGTCCATCCTGACCAT	BT043589.1
	R	CTGAAGAGGTCGGAACACATCTC	
PGC1A^2^	F	GTCAATATGGCAACGAGGCTTC	FJ710605
	R	TCGAATGAAGGCAATCCGTC	
CPT1^3^	F	TCCCACATCATCCCCTTCAACT	AM230810
	R	TGTCCCTGAAGTGAGCCAGCT	
HBB^4^	F	ACAAACGTCAACATGGTCGACTGG	EG897325.1
	R	TCTTTCCCCACAGGCCTACGAT	
HBA^5^	F	AAGGCAGATGTCGTCGGTGCT	CK883845.1
	R	CAGCCCAGTGGGAGAAGTAGGTCTT	
CD8A^6^	F	CGTCTACAGCTGTGCATCAATCAA	AY693391
	R	GGCTGTGGTCATTGGTGTAGTC	
SEC13^7^	F	AGTGGGCCTGTATCAGCGACGT	EG882700.1
	R	ATCACTGCTCGTTCGTCGCTCC	
NDUFB8^8^	F	TCTGTCGCTGGGAGGAGAAGGA	DW532752.1
	R	GTCCAGGCAGGTCCGATACTCTGT	
EF1A^9^	F	CACCACCGGCCATCTGATCTACAA	AF321836
	R	TCAGCAGCCTCCTTCTCGAACTTC	

Complete gene names: ^1^Heat shock protein 70, ^2^Peroxisome proliferator-activated receptor gamma, coactivator 1, ^3^Carnitine palmitoyltransferase 1, ^4^Hemoglobin beta chain, ^5^Hemoglobin alpha chain, ^6^CD8 alpha T cell glycoprotein, ^7^SEC13-like protein, ^8^NADH dehydrogenase (ubiquinone) 1 beta subcomplex, 8, 19 kDa, ^9^Elongation factor 1 alpha.

### Immunofluorescence microscopy

**Figure 1:**
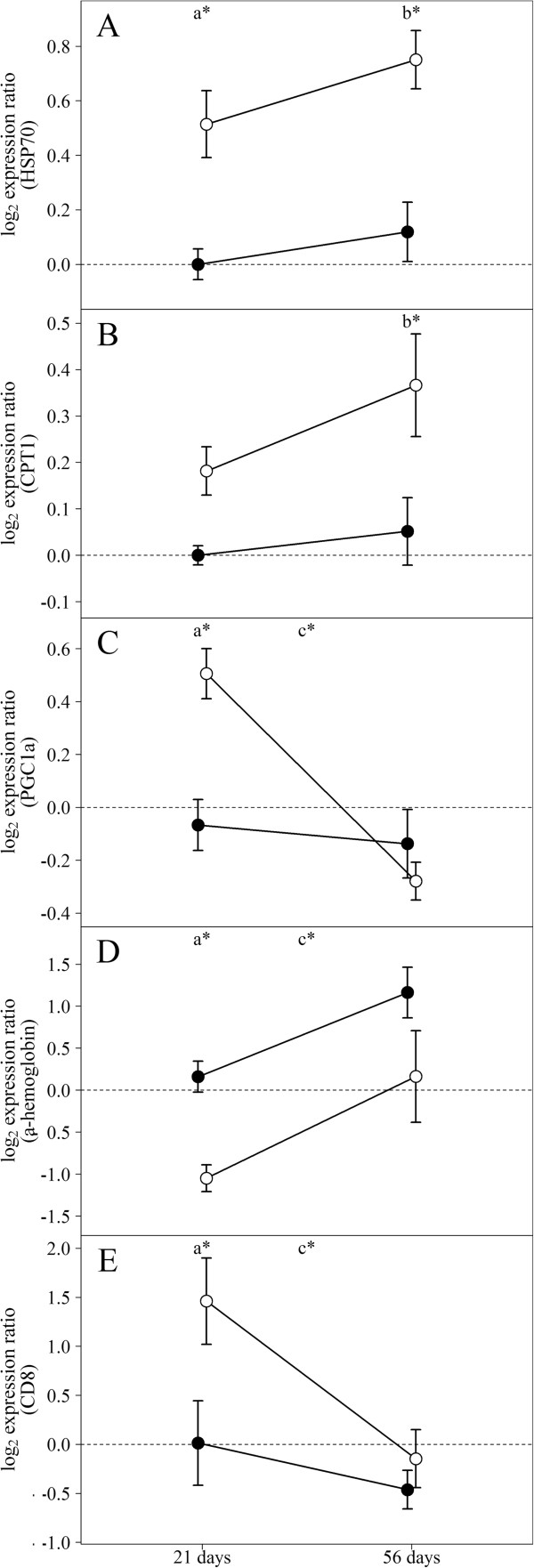
**Cardiac expression of selected genes in fish reared under normal and elevated temperature.** Relative mRNA transcription levels of **A)***heat shock protein 70*, *HSP70*; **B)***carnitine palmitoyltransferase 1*, *CPT1*; **C)***peroxisome proliferator-activated receptor (PPAR)γ coactivator 1α*, *PGC1α*; **D)***α-hemoglobin*; **E)** T cell antigen *CD8 alpha* in fish reared at normal (14°C, filled circles) and elevated (19°C, open circles) temperature for 21 and 56 days. Data are mean log_2_ expression ratio ± SEM relative to the average of normalized controls (14°C, 21 days), real-time qPCR. Statistical differences (adjusted p-value < 0.05; pairwise t-tests of the four groups, N = 9) are indicated between temperatures (21 days: a*, 56 days: b*) and time points for 19°C fish (c*).

**Figure 2:**
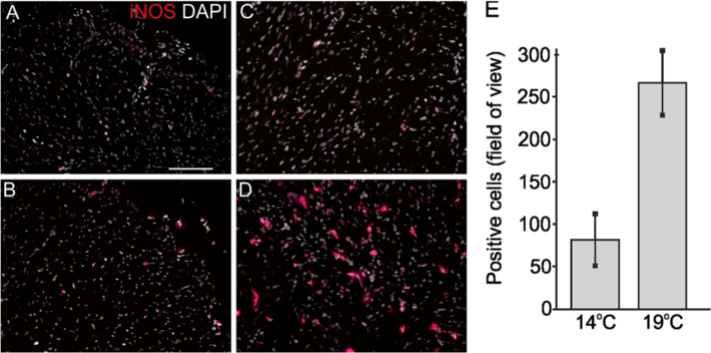
**Inducible nitric oxide synthase (iNOS) expression in cardiac tissues of fish reared under normal and elevated temperature.** Immunofluorescence staining of iNOS (red color) and DAPI nuclear counterstain (white color) in cardiac tissues of fish reared for 56 days at normal temperature (14°C, upper panels) and high temperature (19°C, lower panels). At 14°C iNOS is expressed at low levels and in a few cells in the compact **(A)** and spongy **(B)** myocardium. At 19°C iNOS is expressed in a higher number of cells in the compact myocardium **(C)**, and strong staining of individual myocytes is observed in the spongy myocardium **(D)**. Panels **A**-**D** show one representative micrograph of three sections examined per fish from a total of three fish per temperature group. The 40 μm scale bar in panel **A** applies to all panels in the figure. **E**: Average number of positive cells ± SEM in spongy myocardium, calculated using a larger field of view (25× objective).

**Figure 3:**
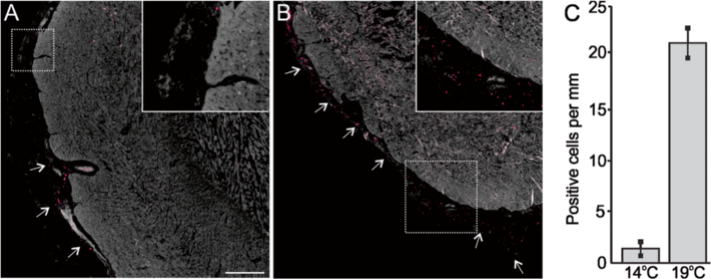
**Vascular endothelial growth factor (VEGF) expression in cardiac tissues of fish reared under normal and elevated temperature.** Immunofluorescence staining of VEGF (red color) in cardiac tissues of fish reared for 56 days at control temperature (14°C, **A**) and elevated temperature (19°C, **B**). **A**: VEGF positive cells (arrows) are mainly located around already existing epicardial vasculature at 14°C. **B**: VEGF positive cells (arrows) are evenly distributed along the entire epicardium at 19°C. Panels **A**-**B** show one representative micrograph of three sections examined per fish from a total of three fish per temperature group, with one representative region per group shown at higher magnification (inset). The 400 μm scale bar in panel **A** applies to both panels in the figure. **C**: Average number of positive cells per mm epicardium ± SEM, calculated using a larger field of view (25× objective).

Hearts were fixed in 4% PFA (paraformaldehyde) and dehydrated in increasing ethanol concentrations prior to paraffin embedding and sectioning (7 μM). After paraffin removal and dehydration, microwave facilitated antigen retrieval was conducted in 10 mM Tris-Hcl (pH = 10) for 10 min. Permeabilization was achieved with 1% Triton X100 for 10 min before 2 hrs blocking in 5% dry milk dissolved in 1 × PBST. Primary antibodies used were rabbit polyclonal antibodies against salmon collagen type I (BioLogo, Kiel, Germany), human/mouse inducible nitric oxide synthase, iNOS (Thermo Fisher Scientific Inc., Rockford, USA) and human/mouse vascular endothelial growth factor, VEGF (147: sc-507, Santa Cruz Biotechnology Inc., Heidelberg, Germany). All were tested for reactivity and specificity in Atlantic salmon [26-28]. Primary antibodies were diluted to a concentration of 5-10 μg/ml in 1 × PBST with 2% dry milk and 1% DMSO. After overnight incubation at 4°C, the sections were washed thoroughly in 1 × PBST and incubated with Alexa conjugated secondary antibodies (Invitrogen) diluted 1:200 for 2 hrs at room temperature. As controls, secondary antibody only was used giving negative results. Final 1 × PBST washes were carried out before mounting and microscopy. All images were captured using a Zeiss Axioplan Z1 and post processed using the Zeiss Axiovison software and Corel Draw. Similar exposure and image manipulation settings were applied to the images to enable comparison between treatments and replicates. A total of three fish (three sections per heart) from each temperature and time point was analyzed. The whole tissue was inspected and one representative image was captured for each section (presented in Figures 1, 2 and 3). For quantification of iNOS expression, the average number of positive cells ± SEM in spongy myocardium (showing the most prominent staining differences between temperature groups) from all fish was calculated with Zeiss Axiovison software from two field of views per fish (25× objective). For quantification of VEGF expression, the average number of positively stained cells ± SEM along the entire epicardium from all fish was similarly calculated (two field of views, 25× objective) and expressed as number of positive cells per mm epicardium. Expression of collagen I between temperature groups was presented as LUT (Look-Up Table) images showing fluorescence intensities of representative sections.

## Results

**Figure 4:**
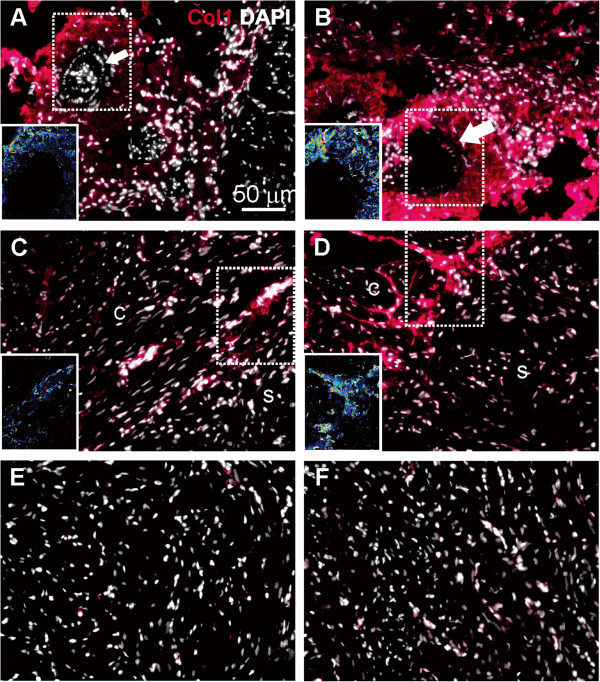
**Collagen I expression in cardiac tissues of fish reared at normal and elevated temperature.** Immunofluorescence staining of collagen I (red color) and DAPI nuclear counterstain (white color) in cardiac tissues of fish reared for 56 days at 14°C (left panels, **A**, **C**, **E**) and 19°C (right panels, **B**, **D**, **F**). **A**: Collagen I is abundant in the epicardium and vasculature (arrow) at 14°C. **B**: Increased signal intensity is observed at 19°C. **C**: At 14°C collagen I is expressed in cell clusters in the compact (c) but not in the spongy (s) myocardium. **D**: At 19°C collagen I is strongly expressed in larger structures resembling connective tissue of the compact (c) but not in the spongy (s) myocardium. **E**-**F**: Cells in the spongy myocardium show weak staining at both temperatures. Fluorescence intensities are shown as LUT (Look-Up Table) images in panels **A**-**D** (inset). Panels show one representative micrograph of three sections examined per fish from a total of three fish per temperature group. The 50 μm scale bar in panel **A** applies to all panels in the figure.

A salmonid cDNA microarray was used to examine temperature effects on cardiac (ventricle) gene expression in adult Atlantic salmon exposed to 19°C (simulating peak summer temperature in Norwegian Atlantic salmon aquaculture) for 21 days (short-term acclimation) and 56 days (long-term acclimation). Temperature affected expression of 11.6% of the genes, of which 52 genes were up-regulated and 156 genes were down-regulated (expression ratio >1.3/log_2_ expression ratio >0.4, 19°C versus 14°C) in at least one time point. These were further grouped according to functional annotation as shown in Table 2, which provided a fundament for supportive analysis on selected genes and proteins using real-time qPCR and immunofluorescence microscopy. Up-regulated features included seven genes involved in cell signaling responses to a diverse array of cellular perturbations such as heat stress and unfolded protein response, among them *hsp47*, *hsp90*, *mapk13*, *junB* (Table 2) and *hsp70* (qPCR; Figure 1A). Immunofluorescence microscopy of iNOS indicated staining of a higher number of cells and at stronger levels in the compact and spongy myocardium after 56 days at 19°C in comparison to control temperature (Figure 2). Immunostaining of VEGF indicated increased and more evenly distributed staining of cells along the epicardium in 19°C fish in comparison to controls, where VEGF was localised in epicardial foci (Figure 3). VEGF labelling was not seen in the compact and spongy muscle layers. Microarray genes coding for structural myocardial proteins showed coordinated down-regulation in fish reared at 19°C compared to 14°C at both time points (Table 2), including different transcripts for myosin, actin and troponin. Among genes regulating extracellular matrix, metalloproteinase genes, involved in degradation of extracellular matrix, was up-regulated whereas several transcripts for collagens were down-regulated with elevated temperature at both time points (Table 2). In contrast, immunostaining of collagen I indicated increased expression in the epicardium and compact myocardium of 19°C versus 14°C fish, while no staining was observed in the spongy layer at either temperature (Figure 4). In regard to energy metabolism, mitochondrial electron transport chain genes such as *NADH-ubiquinone oxidoreductase 15 kDa subunit*, c*ytochromes b/c/c1/c2* and *ATP synthase* were down-regulated with elevated temperature at both time points (Table 2). In addition, genes for cardiac fatty acid oxidation showed significant up-regulation in 19°C fish, including *carnitine palmitoyltransferase* (*CPT*)*1* after 56 days (Figure 1B) and *peroxisome proliferator-activated receptor (PPAR)γ coactivator* (*PGC*)*1α* after 21 days (Figure 1C). Genes involved in oxygen transport and the heme biosynthetic pathway, including several transcripts for *α/β-hemoglobin* and *d-aminolevulinate synthase*, were down-regulated in 19°C fish after 21 and 56 days (Table 2 and Figure 1D). Microarray results indicated that expression of immune-related genes were influenced by the temperature elevation, through down-regulation of genes encoding humoral components of the innate immune system (complement factors, chemokines and receptors, the serine protease activator *cathepsin C-3* and *annexin A1*). In contrast, genes coding for cellular components were up-regulated, such as *CD9 antigen*, *high affinity IgG Fc receptor I precursor*, *tyrosine-protein kinase BTK*, g*amma-interferon inducible lysosomal thiol reductase* (Table 2) and the T cell antigen *CD8 alpha* (Figure 1E).

**Table 2:** Functional categories and genes regulated in Atlantic salmon reared at high temperature for 21 and 56 days identified from microarray analysis

**Probe number**	**Name (best blast hit)**	**Day 21**	**Day 56**
*Unfolded protein response*			
CA356940	Heat shock protein 47 kDa	1,15	1,35
CA373890	Heat shock protein HSP 90-beta-1	0,66	0,67
EST1-3A_F08	Heat shock protein HSP 90-beta-2	0,83	0,80
EXOB1_E03	Eukaryotic translation initiation factor 3 subunit 5	0,55	0,30
EXOB1_E08	Eukaryotic translation initiation factor 3 subunit 6-1	0,44	0,35
CA382570	Mitogen-activated protein kinase 13	0,29	0,57
EST1-3A_H06	Transcription factor jun-B-1	0,43	1,02
CA368716	Membrane-bound transcription factor site 2 protease	0,58	0,36
CA378435	Protein phosphatase 2C delta isoform	0,62	0,23
*Tissue remodeling and cytoskeleton*			
est03c04	Matrix metalloproteinase-9	0,34	0,51
EXOB3_H01	Matrix metalloproteinase-13	0,47	0,81
CA378743	Fibronectin precursor	0,44	0,29
utu04c11	Collagen alpha 1(I) chain-2	-0,32	-0,95
HK0003_C02	Collagen alpha 1(I) chain-1	-0,16	-0,51
utu02b11	Collagen a3(I)-2	-0,29	-0,56
utu02a06	Collagen a3(I)-1	-0,22	-0,41
HKT0001_B03	Alpha 2 type I collagen-1	-0,54	-0,91
HK0003_E07	Myosin light chain 2-1	-0,50	-0,73
utu02c02	Myosin heavy chain 1-1	-0,51	-0,54
utu04f06	Myosin heavy chain 1-2	-0,64	-0,95
HK0002_F05	Myosin heavy chain, fetal	-0,56	-0,50
HK0002_B06	Troponin T-3	-0,81	-0,67
HK0003_D01	Troponin C-1	-0,71	-0,17
HKT0001_E07	Actin, alpha 1	-0.39	-0.71
utu04d04	Actin, alpha 4	-0.13	-0.67
utu04f08	Actin, alpha 5	-0.58	-1.27
HK0003_C08	Parvalbumin alpha-2	-0,78	-0,65
est01e10	Tolloid-like protein (nephrosin)-1	-0,74	-0,47
*Energy metabolism*			
HKT0001_H05	Cytochrome b-3	0,32	0,41
utu02b07	Cytochrome c oxidase subunit II	0,59	0,44
HK0001_G02	ATP synthase beta chain-2	-0,43	-0,71
HK0002_G02	Creatine kinase, sarcomeric mitochondrial precursor	-0,41	-0,63
est03a08	Cytochrome c-1	-0,66	-0,99
HK0003_B03	Cytochrome c-2	-0,91	-1,16
EXOB1_C10	Cytochrome P450 2 K4-2	-0,94	-1,39
HK0002_A12	NADH-ubiquinone oxidoreductase 15 kDa subunit	-0,45	-0,43
*Heme biosynthesis*			
EXOB2_B10	Hemoglobin beta chain Omy 30073	-0,54	-1,64
HST0001_C04	Hemoglobin alpha chain Omy 11839	-0,96	-1,56
EXOB4_H06	Alpha-globin 1-3 Omy 8146	-0,76	-1,41
HST0001_C02	Alpha-globin I-1 Omy 11839	-0,18	-0,97
utu01e09	Embryonic alpha-type globin2 + collagen alpha 2(1)	-0,43	-0,68
HST0001_D08	Beta-globin Omy 9744	-0,79	-1,06
est01g04	5-aminolevulinate synthase	0,08	-0,52
CA381045	Aminolevulinate, delta-, synthase 1	-0,84	-1,23
HK0001_D09	Cytochrome P450 2 F1	-0,87	-1,15
*Immune response*			
EXOB1_F11	CD9	0,45	0,36
CA388403	CD9-like	0,20	0,54
CA378736	Tyrosine-protein kinase BTK	0,44	0,29
CA382425	B-cell translocation gene 1-2	0,49	0,42
EXOB4_C11	High affinity immunoglobulin γ Fc receptor I precursor	0,47	0,39
CA362806	Gamma-interferon inducible lysosomal thiol reductase	0,51	0,17
CA355488	Tapasin-2	0,40	0,45
CA373659	Mannan-binding lectin serine protease 2-2	0,25	0,46
ENH2_B05	Acute phase protein	0,41	0,53
CA370329	Lysozyme C precursor	0,87	0,61
CA362419	Complement component C6	0,47	0,40
HK0001_F01	Complement factor H-1	-0,82	-0,73
CA370696	Complement control protein factor I-B	-0,71	0,23
EXOB1_E12	Serine protease-like protein-3	-0,34	-0,53
EST1-3A_A09	Serine protease-like protein-2	-0,42	-0,65
EXOB3_B01	Cathepsin C-3	-0,90	-1,30
CA377504	Cold autoinflammatory syndrome 1 protein	-0,81	-0,62
HK0002_G10	T-cell receptor α chain V region HPB-MLT precursor (Fr)	-0,91	-0,52
CA372428	Leukotriene B4 receptor 1	-1,11	-0,82
HK0002_G11	Myristoylated alanine-rich protein kinase C substrate	-1,07	-0,87
EXOB2_G01	Leukocyte cell-derived chemotaxin 2	-0,97	-0,86
CA343700	CXC chemokine receptor transcript variant B	-0,52	-0,88
CA361151	Annexin A1-2	-0,58	-0,47

Expression values are log_2_-expression ratios (19°C versus 14°C) from analysis of pooled heart samples (per temperature treatment: 3 fish per triplicate tanks, n = 9).

## Discussion

Seasonal fluctuations in seawater temperature are naturally occurring in the aquaculture of Atlantic salmon. Particularly in the summer months, western and southern regions of Norway experience temperature increments above thermal preference (15°C [3,29,30]) peaking around 18-20°C. In a recent study we observed significantly reduced feed intake, growth performance and endogenous energy storage in large (~2 kg) Atlantic salmon after long-term exposure to 19°C under controlled conditions [19]. This poor performance was linked to suppressed endocrine appetite regulation, leading to a negative energy homeostasis with depleted lipid stores in muscle and whole carcass. Based on these findings and the same experimental fish, the current study aimed to further understand how the chronic temperature elevation affected molecular processes at the levels of gene and protein expression in the heart, as a key organ for thermal plasticity and acclimation in salmonids [31,32].

Among genes that were strongly up-regulated with elevated temperature, it was not surprising to find several chaperones and other genes involved in the unfolded protein response. This included three heat shock proteins (HSPs), which are among the most studied proteins in the general response to a variety of stressors and perturbations in mammals and fish [33]. Although their use as suitable indicators of stressed states in fish has been disputed [34], synthesis of HSPs to maintain proper folding/refolding of proteins has been shown highly expensive for the organism [35]. In spite of a severely compromised energy homeostasis in the experimental fish, the sustained induced transcription of HSPs over time could indicate a need for chaperone activity and the unfolded protein response during cardiac acclimation to the elevated temperature.

The nitric oxide synthase system is an important inter- and intracellular regulator of mechanical performance and oxygen supply in the fish heart [15], and iNOS is exclusively expressed in ventricular cardiomyocytes under basal condition and after LPS stimulation [36]. Although the physiological and pathophysiological regulation of the NOS/NO system is very complex and only partly understood in fish, studies have shown that NOS influences cardiac inotropy (i.e. cardiac output) of salmon [37] and the red-blooded icefish *T. bernacchii*[36], which was also sensitive to acute temperature change [16]. Our results showing increased immunofluorescence staining of iNOS in compact and spongy myocardium may be a further indication of an involvement in the thermal acclimation or response to increased temperature. NO has also been shown to induce vasodilation and reduce coronary resistance under hypoxia in salmonids [38]. In this process, VEGF is another important regulator which promotes proliferation and migration of endothelial cells in the formation of new blood vessels under cardiac physiological and pathological conditions [39]. Whether the induced expression of VEGF and iNOS in our study reflected increased vascularization and/or vasodilation to compensate for increased oxygen demand with elevated temperature should be subject to further study. In this regard, the lower abundance of several transcripts for heme biosynthesis and ATP production/energy metabolism after short- and long-term exposure to elevated temperature could also indicate that oxygen transport/uptake and possibly aerobic metabolism was affected.

A fundamental mode for increasing oxygen carrying capacity in vertebrates is by increasing the cardiac output. In salmonids, several studies have suggested that a limitation at the level of the heart is the primary cause of limited oxygen supply with increasing temperature because maximum cardiac output fails to increase above thermal optimum [13]. Furthermore, acclimation to high temperatures has been associated with increased thickness of the compact myocardium, which is assumed to compensate for the decreased power-generating ability [14] or simply reflecting an increased activity level at higher temperatures [40]. A recent study with warm-acclimated rainbow trout also reported that the increased thickness of the compact layer was associated with reduced connective tissue, however this was only observed for male and not female fish [10]. Correspondingly, we observed lower abundance of collagen mRNA with high temperature, however effects of sex was not determined. On the contrary, immunostaining demonstrated increased deposition of collagen I in large bundles exclusively in the compact myocardium at high temperature. This discrepancy between protein and transcript levels of collagen could be methodology based, since gene expression was measured in total RNA extracted from the whole myocardium (both spongy and compact layers) whereas immunofluorescence detection of collagen showed increased staining with temperature only in the compact myocardium, which typically comprises 30-50% of the heart in athletic fishes [7]. In fact, Klaiman *et al.*[10] reported reduction in muscle bundle area in spongy myocardium with warm acclimation, thus lower collagen gene expression levels could simply reflect altered proportions of compartments with increased temperature. Pointing in the same direction, we also observed substantially lower transcript levels of several cytoskeleton genes including actin, myosin and troponin after short- and long-term acclimation. In view of the severely compromised growth performance and energy homeostasis of the fish in this study after long-term high temperature, implications of the compact myocardium increase in collagen I protein expression on cardiac function and possibly fibrosis should be further studied. Reduced mitochondrial capacity above thermal optimum has been demonstrated for fish [41]. Interestingly, we found significantly higher mRNA levels of CPT1 (after 56 days) and PGC1α (after 21 days) in fish kept at 19°C. It could be speculated whether this was a tissue response in order to balance the overall energetic status, a notion supported from reduced hepatic beta-oxidation in the same fish [19].

High temperature affected cardiac expression of important immune-related genes. The salmon heart is an immune-relevant organ in the sense that it represents a major replication site for several viral pathogens of high importance [17,18]. Common for these diseases is the strong inflammation of the myocardium which coincides with a strong activation of a CD8 T cell response [18,23,42]. Consequently, given the temperature-induced gene expression of cellular immune components including CD8 alpha in the present study, it may be speculated if high seawater temperatures can further elevate myocardial inflammation during infection and thus represent a relevant risk factor affecting disease outcome. It has been proposed that low environmental temperature diminishes the humoral immune response, since primary antibody response has been found to be either dampened or retarded at lower temperatures [43,44]. Here, we report that expression of some genes involved in the innate humoral immune response were down-regulated with high temperature. At the same time, a suite of genes representing cellular components were up-regulated, including some involved in B- and T-cell development. Our understanding of immunological effects of elevated temperatures in fish is far from complete, but studies have shown significant hematological modulation in response to thermal acclimation in salmonids [45-47]. A study with rainbow trout reported induced complement lytic activity and opsonization capacity with increased temperature in rainbow trout [48], which could agree with our finding of temperature-induced gene expression of *complement C6*, a part of the membrane attack complex. Another aspect relates to whether our observed regulation of cardiac innate immune response genes is ascribed to the temperature increase *per se* or (re)allocation of resources in view of the negative growth performance and energy homeostasis of the fish. This is a subject that should deserve attention in future research.

## Conclusions

This study provides new knowledge on the molecular responses of cardiac tissues during long-term exposure to elevated seawater temperature reflective of the peak summer temperatures experienced in Atlantic salmon aquaculture. We report temperature-induced changes in expression of genes and proteins indicating that the unfolded protein response, vascularization and remodeling of connective tissue as well as altered innate immune responses were affected during cardiac acclimation to elevated temperature.

## Competing interests

The authors declare that they have no competing interests.

## Authors’ contributions

SMJ designed and performed the microarray studies together with AK, and participated in samplings, data interpretation and wrote the manuscript together with VC, who also performed qPCR gene expression studies together with GT. AK were responsible for microarray data processing and analysis as well as revision of the manuscript. JT conducted immunofluorescence microscopy. HT obtained funding, planned and coordinated the experimental fish study and participated in samplings together with EMH, TJH, SS and OB, and also revised the manuscript. All authors read and approved the final manuscript.
